# *Ex Vivo* Response to Histone Deacetylase (HDAC) Inhibitors of the HIV Long Terminal Repeat (LTR) Derived from HIV-Infected Patients on Antiretroviral Therapy

**DOI:** 10.1371/journal.pone.0113341

**Published:** 2014-11-19

**Authors:** Hao K. Lu, Lachlan R. Gray, Fiona Wightman, Paula Ellenberg, Gabriela Khoury, Wan-Jung Cheng, Talia M. Mota, Steve Wesselingh, Paul R. Gorry, Paul U. Cameron, Melissa J. Churchill, Sharon R. Lewin

**Affiliations:** 1 Department of Infectious Diseases, Monash University, Melbourne, Victoria, Australia; 2 Centre for Biomedical Research, Burnet Institute, Melbourne, Victoria, Australia; 3 Department of Microbiology and Immunology, University of Melbourne, Melbourne, Victoria, Australia; 4 South Australian Health and Medical Research Institute, Adelaide, Australia; 5 Infectious Disease Unit, Alfred Hospital, Melbourne, Victoria, Australia; 6 Department of Microbiology, Monash University, Clayton, Victoria, Australia; 7 Department of Medicine, Monash University, Clayton, Victoria, Australia; 8 Peter Doherty Institute, Melbourne University, Melbourne, Victoria, Australia; George Mason University, United States of America

## Abstract

Histone deacetylase inhibitors (HDACi) can induce human immunodeficiency virus (HIV) transcription from the HIV long terminal repeat (LTR). However, *ex vivo* and *in vivo* responses to HDACi are variable and the activity of HDACi in cells other than T-cells have not been well characterised. Here, we developed a novel assay to determine the activity of HDACi on patient-derived HIV LTRs in different cell types. HIV LTRs from integrated virus were amplified using triple-nested Alu-PCR from total memory CD4^+^ T-cells (CD45RO+) isolated from HIV-infected patients prior to and following suppressive antiretroviral therapy. NL4-3 or patient-derived HIV LTRs were cloned into the chromatin forming episomal vector pCEP4, and the effect of HDACi investigated in the astrocyte and epithelial cell lines SVG and HeLa, respectively. There were no significant differences in the sequence of the HIV LTRs isolated from CD4^+^ T-cells prior to and after 18 months of combination antiretroviral therapy (cART). We found that in both cell lines, the HDACi panobinostat, trichostatin A, vorinostat and entinostat activated patient-derived HIV LTRs to similar levels seen with NL4-3 and all patient derived isolates had similar sensitivity to maximum HDACi stimulation. We observed a marked difference in the maximum fold induction of luciferase by HDACi in HeLa and SVG, suggesting that the effect of HDACi may be influenced by the cellular environment. Finally, we observed significant synergy in activation of the LTR with vorinostat and the viral protein Tat. Together, our results suggest that the LTR sequence of integrated virus is not a major determinant of a functional response to an HDACi.

## Introduction

Despite the substantial reduction in morbidity and mortality following combination antiretroviral therapy (cART), current treatments do not cure HIV and treatment is required life-long. The major reason why cART cannot cure HIV is the persistence of HIV in resting memory and naïve CD4^+^ T-cells [1], [2]. One strategy currently being pursued to eliminate latently infected cells is to stimulate virus production from latency [3].

Histone deacetylase inhibitors (HDACi) can activate HIV production efficiently in nearly all latently infected cell lines [4]–[9]. In contrast, in primary CD4^+^ T-cell models of latency, the capacity of an HDACi to activate virus production from a latent provirus is variable –depending on the model used [10]. Using resting CD4^+^ T-cells from HIV-infected patients on cART *ex vivo*, the HDACi vorinsotat induced virus production in 50–80% of patients, in both the absence [11] or presence [8], [12] of activated feeder cells. More recently, where virus production from patient cells was measured by RT-PCR in the absence of feeder cells, there was minimal virus production with vorinostat compared to the more potent HDACi romidepsin [9]. When using a reporter cell line to measure infection, other studies have shown minimal virus production following stimulation with all HDACi relative to maximal stimulation with a mitogen such as PMA and ionomycin [13]. Finally, when vorinostat and panobinostat were given to HIV-infected patients on ART, a variable response in activation of latent HIV has been observed in multiple studies [12], [14], [15]. Taken together these studies demonstrate a far more variable response of patient derived cells to treatment with HDACi *ex vivo* compared to models that are infected with laboratory strains of HIV.

It is likely that latent proviruses *in vivo*, may have variable sensitivity to stimulation with an HDACi. Factors such as sequence of the HIV LTR, the surrounding chromatin environment, the specific integration site or the cellular environment may all potentially play a role. Here, we found that the HIV LTR sequence isolated from T cells was not a factor in determining an *ex vivo* response to HDACi but that the capacity of an HDACi in inducing HIV transcription was dependent on the cell type examined with maximal LTR transcription observed in an epithelial cell line. Finally, the potency of the HDACi vorinostat was significantly enhanced in the presence of the viral protein Tat.

## Materials and Methods

### Patient recruitment

HIV-infected, cART naïve patients (n = 4) who were initiating cART were recruited at the Alfred Hospital, Melbourne. This was a sub-study of a previously reported prospective observational study [2]. Fifty millilitres of blood were collected at baseline and at 6, 12, 18, 24, and up to 60 months after initiation of cART. The parent study and sub-study were both approved by the Alfred hospital ethics committee (114/05) and written informed consent was obtained from all participants.

### Isolation of the integrated HIV LTR from CD4^+^ T-cells

Total memory T-cells from HIV-infected patients, defined as CD4^+^CD45RO^+^CD28^+^, were isolated by flow cytometry sorting using anti-CD4-fluorescein isothiocyanate (FITC), anti-CD28-PE-cyanine dye (Cy5) and anti-CD45RO-allophycocyanin (APC; Becton Dickinson, San Jose, CA). Cells were lysed for PCR analysis using PCR lysis buffer (0.002% Triton X100, 0.002% SDS, 100 mM Tris.HCl (pH 8), 1 mM EDTA with freshly added Proteinase K 0.8 mg/ml). The DNA lysate was serially diluted using 1 in 10 dilutions and between 12 and 24 replicates from each dilution were added to a first round PCR mix containing 0.2 µM of Alu1, Alu2 and 5′LTRf2 primers (Table 1) in ImmoMix PCR premix (Bioline, London, UK). The PCR was performed using conditions described in Table 1. Two microliters of the first-round product were then added to a second-round PCR mix containing 0.2 µM 5′LTRf2 and 3′nLTR#2 primers in ImmoMix PCR premix (Table 1). Subsequently, two microliters of the second-round product were then added to a third-round PCR mix containing 0.2 µM 5′KpnI-LTRf3 and 3′nLTR#3 primers in ImmoMix PCR premix (Table 1). The resulting products of the first round PCR were Alu-LTR, LTR-Alu, Alu-Alu with varying length, depending on the distance of the integrated LTR to the closest Alu. The second and third round PCRs then preferentially amplify the integrated LTR sequence. PCR products were analysed by 1% agarose gel electrophoresis. The dilution that yielded a PCR product in <30% of replicates, was assumed to contain one amplifiable template per reaction more than 80% of the time, according to a Poisson distribution [16]. Using ACH2 cells (NIH AIDS Reagent Program [17]), a T-cell line that contains a single integrated copy of HIV, we determined that the lower limit of detection was <2 copies of HIV DNA per well. PCR products were sequenced and hypermutated LTRs (G->A) were identified using the software Hypermut (http://www.hiv.lanl.gov/content/sequence/HYPERMUT/hypermut.html). Hypermutated LTRs were removed from further analyses. A phylogenetic tree was constructed using the neighbour-joining method with CLC Workbench software (Version 6, CLC Bio, Aarhus, Denmark), with bootstrapping resampling done with 100 replicates. The consensus NL4-3 sequence was used for comparison with patient samples.

**Table 1:** Primers and triple-nested Alu-LTR-PCR conditions.

	Primer	Sequence	PCR Condition
Round 1	Alu1	TCCCAGCTACTGGGGAGGCTGAGG	94°C for 10 minutes, 20 cycles of (94°C for 15 seconds, 56°C for 15 seconds and 72°C for 2 minutes), final extension at 72°C for 7 minutes
	Alu2	GCCTCCCAAAGTGCTGGGATTACAG	
	5′LTRf2	GCCACTTTTTAAAAGAAAAGGGGGACT	
Round 2	5′LTRf2	GCCACTTTTTAAAAGAAAAGGGGGACT	94°C for 10 minutes, 35 cycles of (94°C for 15 seconds, 51°C for 15 seconds and 72°C for 1 minutes), final extension at 72°C for 7 minutes
	3′nLTR#2	AAAAGGGTCTGAGGGATCTCT	
Round 3	5′KpnI-LTRf3	GGGGTACCAAAGGGGGACTGAAGGGCTAATTC	94°C for 10 minutes, 35 cycles of (94°C for 15 seconds, 52°C for 15 seconds and 72°C for 1 minutes), final extension at 72°C for 7 minutes
	3′nLTR#3	ACGGGCACACACTACTTGAA	

### Cells lines and cytotoxicity assay

The SVG astrocyte cell line [18] was cultured in Minimum Essential Medium (MEM) with Earle's salts supplemented with 10% heat-inactivated fetal bovine serum (HI-FBS), 1× penicillin-streptomycin-glutamine (PSG) (Invitrogen, Carlsbad, CA). The HeLa cell line (ATCC) was cultured in MEM containing 10% HI-FBS and 1× PSG.

Each cell line was treated with varying concentrations of trichostatin A (TSA; Sigma), vorinostat [suberoylhydroxamic acid (SAHA)], panobinostat (LBH589) and entinostat (MS-275; all from Selleck Chemicals, Houston, TX) for 24 hours and toxicity was assessed with the MTS colorimetric assay according to manufacturer's instructions (Promega, Madison, WI). Experiments were performed in triplicate and the cytotoxic concentration 50 (CC_50_) values were generated using GraphPad Prism software (Version 6, Graphpad, La Jolla, CA).

### Cloning of HIV LTR into pCEP4

**Figure 1:**
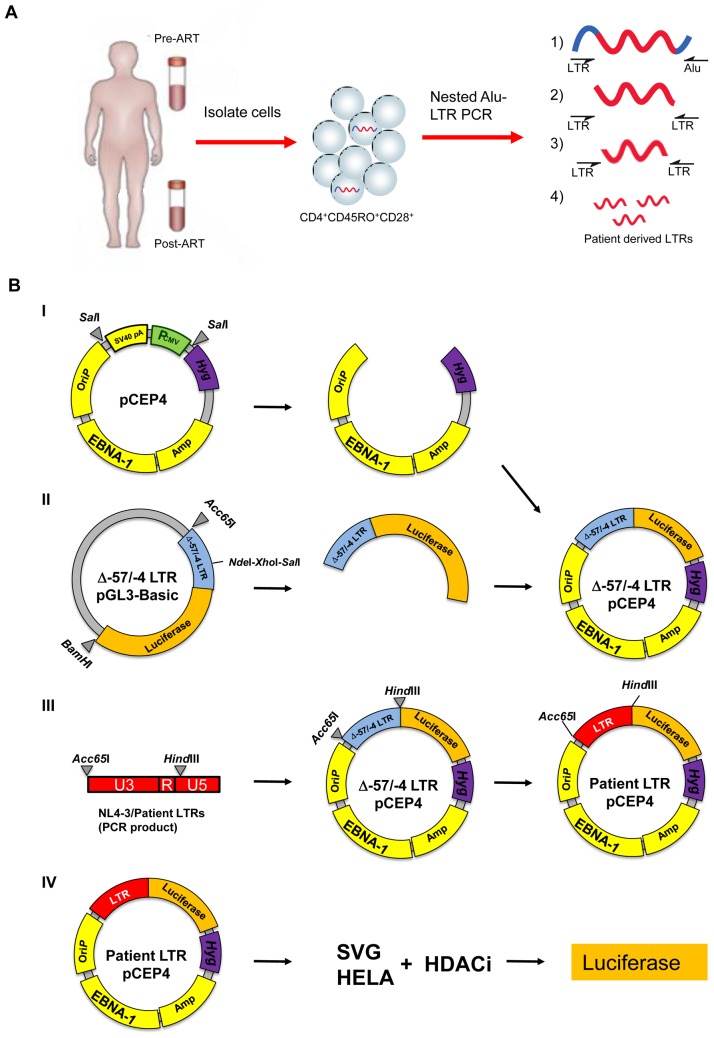
Cloning of patient-derived HIV LTRs into pCEP4. (A) Total memory T cells from HIV-infected individuals were isolated from blood collected prior to or after receiving cART. The integrated HIV LTRs from these cells were isolated by triple nested Alu-LTR PCR. (B)(I) The plasmid pCEP4 was digested with *Sal*I to remove the entire PCMV promoter region and the SV40 poly A sequences. (II) DNA sequence of the Δ-57/-4 HIV LTR and the luciferase gene was generated by digestion of the Δ-57/-4 LTR pGL3-Basic vector [20] and ligated into pCEP4. (III) Patient-derived HIV LTRs were cloned into the Δ-57/-4 LTR-pCEP4 vector using the *Acc*6*5*I and *Hind*III sites. (IV) Patient LTR pCEP4 was transfected into SVG and HeLa cell lines; the activity of various HDACi on LTR transcription was measured by quantification of luciferase activity.

The plasmid pCEP4 (Invitrogen) was digested with *Sal*I (NEB Biolabs, Ipswich, MA) to remove the entire existing promoter region, the multiple cloning site and the SV40 poly A sequence (Fig 1). The plasmid was then blunt ended and a fragment of the Δ-57/-4 HIV [19] LTR-pGL3 plasmid spanning the entire LTR and including the luciferase gene [20] was digested with *Acc*65I/*BamH*I and cloned into pCEP4. To clone the patient LTRs into pCEP4, patient-derived LTRs and the Δ-57/-4 HIV LTR-pCEP4 vector were both digested with *Acc*65I and *Hind*III according to the manufacturer's instructions (NEB Biolabs). The digested LTRs and pCEP4 vector were ligated using T4 DNA Ligase (NEB Biolabs). Correctly inserted clones were initially screened by colony-PCR and later confirmed by restriction digest using *Sal*I (NEB Biolabs).

The pCEP4 plasmid contains an Epstein-Barr virus nuclear antigen protein (EBNA-1) to allow for extrachromosomal replication in mammalian cells [21]. Importantly, any genes cloned into pCEP4 are susceptible to post-transcriptional modifications such as methylation [21], [22], which is important for our study.

### Transfection of cells with pCEP4

HeLa (3,000 cells/well) or SVG (5,000 cells/well) cells were seeded overnight into a 96-well flat-bottom plate. The following day, media was replaced with 100 µL of cell media without antibiotics and transfected with 50 µL of pre-mixed Lipofectamine 2000 (Invitrogen) contain 200 ng/well of patient-derived or NL4-3 LTR-pCEP4 according to the manufacturer's instructions (Invitrogen). In some experiments, cells were also co-transfected with 4 ng/well of pTargeT-HxB2-Tat vector [20]. After 4 hours, cell culture media was replaced with fresh culture media containing antibiotics and incubated for another 20 hours. Subsequently, various doses of panobinostat, trichostatin A, vorinostat and entinostat; or phorbyl myristoacetate (PMA [20 ng/ml]; Sigma St. Louis, MO) were added for 24 hours. Cells were then lysed in 1× Luciferase Assay Buffer (Promega, Madison, WI) and the transcriptional activity of the LTR was measured by quantifying luciferase expression using the Luciferase Assay System (Promega) according to manufacturer's instructions. Luminescence was measured using a FLUOStar Optima microplate reader (BMG Labtech, Ortenberg, Germany).

To determine the transfection efficiency, HeLa or SVG were transfected as above with pEGFP-N1 (Clontech, Mountain View, CA), a GFP-expressing plasmid, and the percentage of cells expressing GFP was analysed 24 hours after transfection.

### Quantification of HDAC proteins

To determine the expression level of HDAC 1, 2, 3 and 4 proteins in HeLa and SVG, five million cells were lysed in RIPA lysis buffer (Thermo Scientific, Rockford, IL), containing 1% Halt protease inhibitor cocktail (Thermo Scientific). Protein concentration was determined using a Bradford protein assay (Bio-Rad, Hercules, CA). Twenty microgram of total proteins were loaded onto a 10% SDS PAGE, transferred onto a PVDF membrane and sequentially probed with rabbit anti-HDAC1, 2 and 4 and goat anti-HDAC3 (Santa Cruz Biotechnology, Dallas, TX). HDAC proteins were detected using Alexa Fluor 680-conjugated goat anti-rabbit or donkey anti-goat secondary antibodies (Cell Signaling Technology, Danvers, MA) and imaged on an Odyssey fluorescent reader (LI-COR, Lincoln, NE).

### Statistical analysis

The potency of HDACi in each cell line was analysed using one-way analysis of variance (ANOVA) with Tukey post-test. The potency of HDACi between different cell lines was measured using a Student's *t*-test. The synergistic effects of vorinostat with PMA or Tat co-transfection was analysed using a Student's *t*-test where the combined effect of vorinostat + PMA or vorinostat + Tat was compared to the sum effect of the individual treatments (GraphPad Prism 6, LA Jolla, CA).

## Results

### HIV LTRs sequences isolated from memory CD4^+^ T-cells show no significant changes following cART

**Figure 2:**
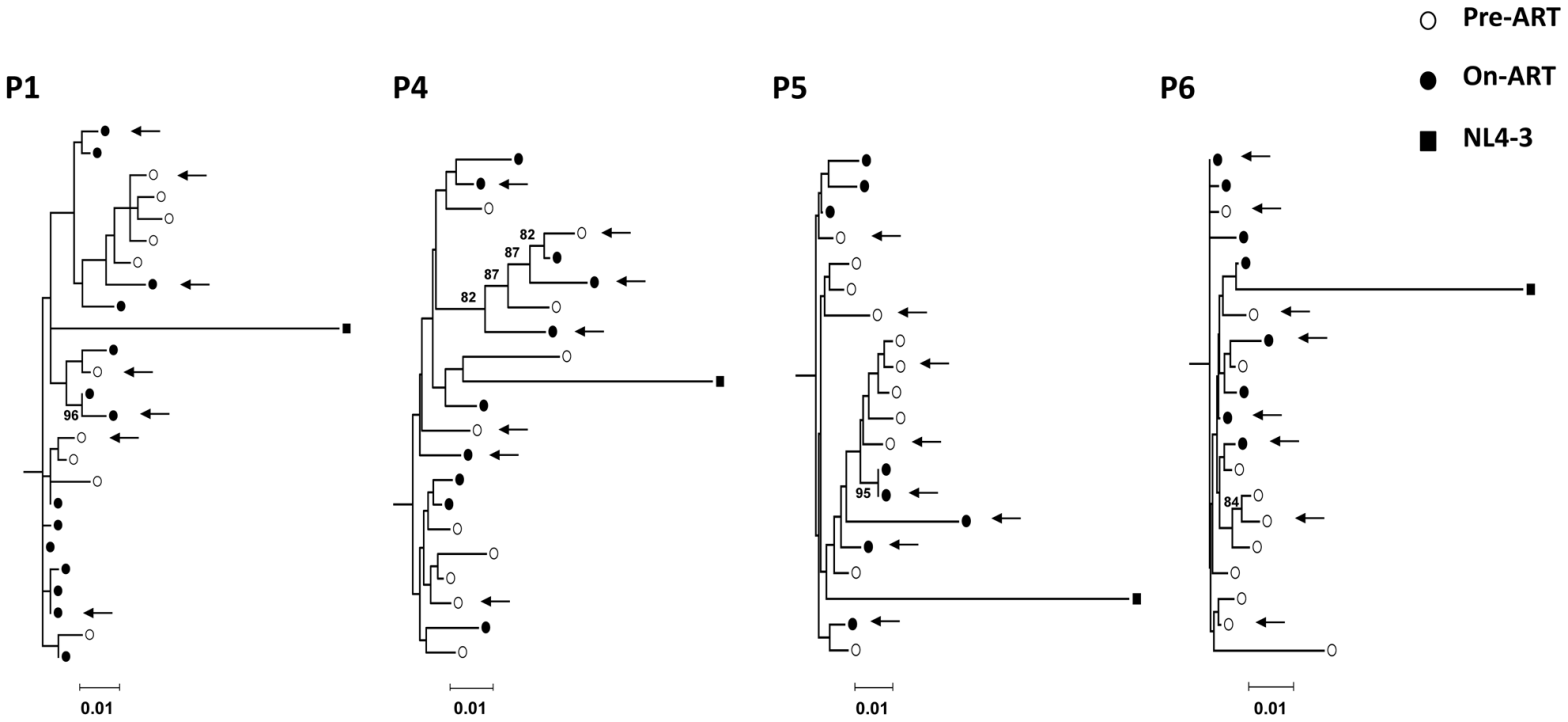
Phylogenetic analyses of DNA sequences derived from integrated virus in CD4^+^ memory T-cells. Phylogenetic trees were constructed using a neighbour-joining method with sequences from nucleotide 6 to 548 of the LTR derived from memory CD4^+^ T-cells prior to the initiation of cART (*open symbols*), after at least 18 months of cART (*closed symbols*) in four participants and the consensus sequence from NL4-3 (square symbol). Arrows indicate clones selected at random for cloning into pCEP4. Scale-bars indicate genetic distance (e.g., 0.01 = 1% genetic distance). Bootstrap values of >75 are shown on branches. All hypermutated clones (*P*<0.05 analysed on Hypermut V2.0) were excluded from the analysis.

To investigate whether there was selection or evolution of the HIV LTR following suppressive cART, integrated HIV LTR from memory CD4^+^ T-cells from HIV infected patients prior to and after suppressive cART were cloned and sequenced. The clinical details of these patients (n = 4) are summarised in Table 2. Sequence analysis of the integrated HIV LTRs showed no compartmentalisation between viral sequences in memory CD4^+^ T-cells prior to and following 18–24 months (Fig 2, P4–6) or up to 60 months of suppressive cART (Fig 2, P1).

**Table 2:** Patient characteristics.

Patient ID	Age* (yrs)	Study sample (time on ART)	VL (RNA copies/ml)	CD4 count (cell/µl)
P1	43	BL	3700	27
		(60 months)	<50	183
P4	71	BL	100000	71
		(18 months)	<50	397
P5	41	BL	100000	118
		(24 months)	<50	792
P6	41	BL	71700	129
		(18 months)	<50	297

BL, Baseline; VL, viral load in plasma; * age at recruitment ie BL.

### Patient-derived HIV LTRs are responsive to reactivation by all HDACi

**Figure 3:**
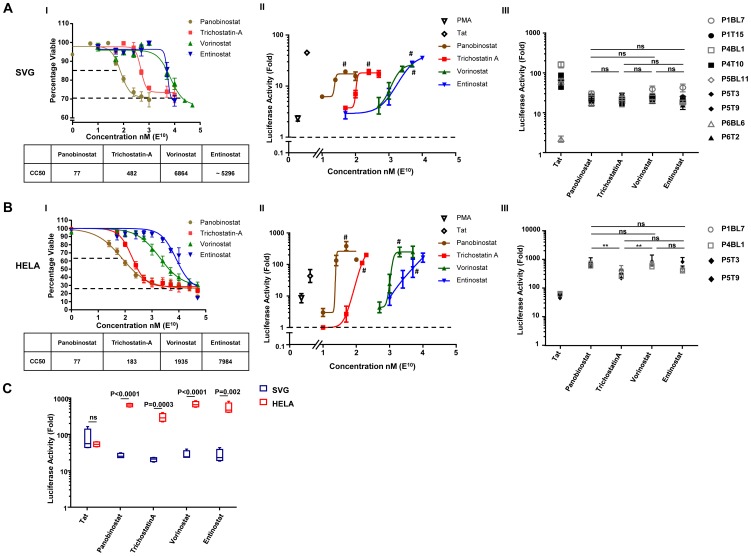
*Ex vivo* response of patient-derived HIV LTRs to HDACi in A. SVG and B. HeLa cell lines. (I) Each cell line was incubated with different concentrations of HDACi for 24 hr and toxicity was measured by the MTS assay. The cytotoxic concentration 50 (CC_50_) for each drug is shown. (II) SVG or HeLa cells were transiently transfected with the wild-type NL4-3 LTR- pCEP4 and treated with various concentrations of HDACi for 24 hr. Co-transfection with Tat (4 ng) or incubation with PMA (20 nM) were used as positive controls. The activity of the LTR was measured as the fold change in luciferase compared to the untreated sample. # indicates doses of individual HDACi that were closest to the CC_50_ and induced the largest fold change increase in luciferase activity. (III) Luciferase expression following transfection of pCEP4 containing LTR sequences isolated from total memory CD4^+^ T-cells prior to or after cART and treated with the optimal dose of HDACi. (C) Comparison of the luciferase expression in HeLa (red) and SVG (blue) following transfection of pCEP4 containing LTR sequences from (B III) and treated with the optimal dose of HDACi. Boxes represent the median, 25th and 75th percentiles and error bars the 10th and 90th percentiles. Ns  =  not statistically significant.

To characterise the *ex vivo* response of patient derived HIV LTR to HDACi, we first determined the cytotoxicity of each HDACi in each cell line. In the SVG astrocyte cell line, cytotoxicity assays showed that the HDACi panobinostat (CC_50_: 77 nM) was the most toxic followed by trichostatin A (CC_50_: 482 nM), entinostat (CC_50_: ∼5296 nM) and vorinostat (CC_50_: 6864 nM) (Fig 3A–I).

We then determined the dose response of individual HDACi on the HIV LTR using the pCEP4 plasmid that contained wild-type NL4-3 LTR. The pCEP4 plasmid forms a mini chromatin structure following transfection [23], which does not integrate into the host genome. This approach therefore allowed us to examine the effects of HDACi independent of integration site.

Following transfection of NL4-3 LTR-pCEP4 into SVG cells and treatment with various doses of HDACi, we quantified luciferase activity. Treatment with PMA (20 ng/ml) and co-transfection with Tat (4 ng) were used as positive controls and induced an increase in luciferase activity of 2.3±0.3 and a 45.0±1 fold above the media-treated sample, respectively. Panobinostat (50 nM), trichostatin A (250 nM), vorinostat (5 µM) and entinostat (5 µM) induced an increase in luciferase activity above the media-treated sample of 17.4±2.6, 17.7±3.3, 25.8±1.7 and 26.8±3.2 fold respectively (Fig 3A–II; n = 3). These concentrations of HDACi were used in subsequent experiments in SVG as they were shown to induce the maximal response from NL4-3 LTR-pCEP4 but were below the CC_50_ value.

Patient-derived LTRs isolated prior to and following cART were ligated into pCEP4 as described above. We found that the patient derived LTRs had similar sensitivity to HDACi as seen with NL4-3 LTR-pCEP4 with a mean ± SE fold increase in luciferase above untreated control following panobinostat (23.8±2.7), trichostatin A (22.4±0.8), vorinostat (26.4±3) and entinostat (25.9±3.9) (Fig 3A–III, n = 9). There was no significant difference in the maximum fold luciferase induction between HDACi at the concentrations tested. LTRs derived from patients while on suppressive cART were also sensitive to reactivation by all HDACi (Fig 3A–III). To confirm that we were not missing subtle differences between patient-derived LTRs following stimulation with lower concentration of HDACi rather than maximal stimulation, we also evaluated the response of three patient derived LTRs to a wide range of HDACi concentrations and again found no significant differences between the patient derived LTRs which all had similar EC50 to the wild type LTR (Figure S1).

Most LTRs showed a greater responsiveness to Tat than to treatment with HDACi (range 2.2-160 fold above untreated control). We identified one clone that was highly sensitive to reactivation by Tat (P4BL1) and a clone that was resistant to Tat-mediated transactivation (P6BL6). There were no obvious mutations of the LTR at the NF-kB, SP1 or TATA sites [24], [25] that may have rendered this clone resistant to tat-mediated transactivation.

The same experiments were then performed in HeLa cells (Fig 3B–I to III) where the CC_50_ for each HDACi was similar to what we found with SVG (Fig 3B–I). In the HeLa cell line, the maximal fold induction of luciferase from patient-derived LTRs (n = 4) by panobinostat (622±35 fold increase) and vorinostat (660±59 fold increase) were significantly greater compared to trichostatin A (288±37 fold increase; p = 0.004), but was not significantly greater than entinostat (522± fold increase) (Fig 3B–III).

### Significantly greater response of the HIV LTR to HDACi in the HeLa compared to SVG cell lines

We observed similar levels of luciferase in HeLa compared to SVG following stimulation with Tat (approximately a 40 fold increase) but significantly higher levels of luciferase activity following stimulation by each of the HDACi in HeLa (Fig 3C). The differences in the maximum fold induction of HDACi in SVG and HeLa cells were unlikely to be attributed to the expression of the HDAC proteins 1–4 which were similar in the two cell lines (Figure S2A) or efficiency of transfection given that we observed similar transfection efficiency in both cell types when using an expressed green fluorescent protein (GFP) reporter plasmid (Figure S2B).

### Vorinostat synergises with Tat to increase transcription of HIV LTRs

**Figure 4:**
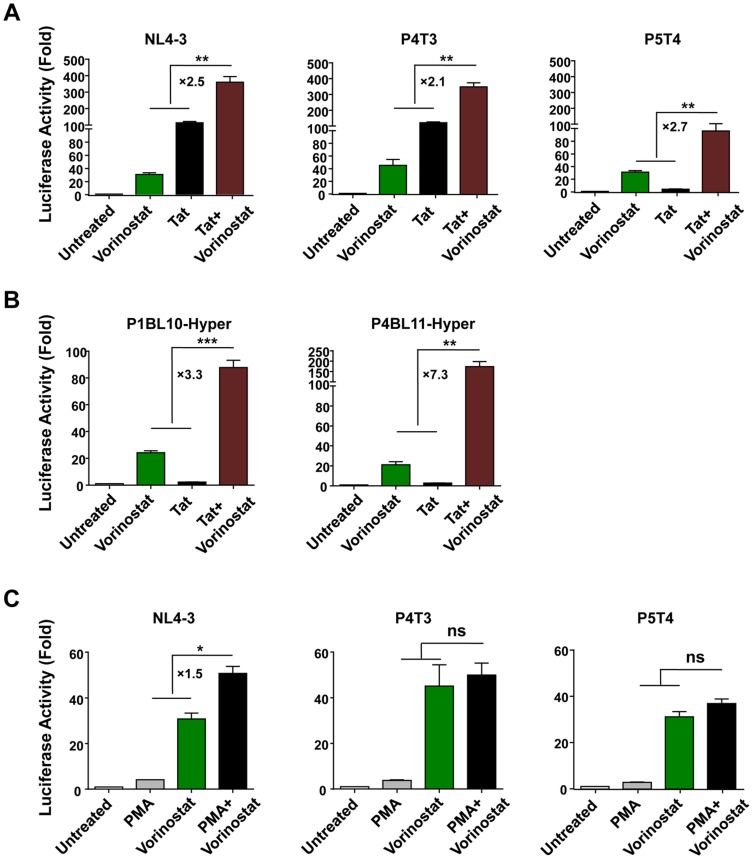
Vorinostat synergises with Tat and not PMA to increase transcription of the HIV LTR. SVG cells were transiently transfected with pCEP4 plasmid containing either NL4-3 or patient-derived HIV LTRs and luciferase activity was quantified following treatment with vorinostat (5 µM) and co-transfection with Tat using (A) non-mutated or (B) hypermutated (*P*<0.05 analysed on Hypermut V2.0) patient-derived HIV LTRs. (C) luciferase activity was quantified following stimulation of non-mutated patient-derived LTRs with vorinostat and PMA (20 ng/ml). Error bars represent standard error of the mean of three independent experiments. * *P*<0.05, ** *P*<0.01 and *** *P*<0.001. ns  =  not statistically significant.

Given that the transactivation activity of Tat is tightly regulated by post-translational modification such as acetylation and methylation [26]–[28], we next investigated whether there was a synergistic response of the LTR to Tat and an HDACi. Using NL4-3 LTR-pCEP4 transfected into the SVG cell line, we showed that vorinostat significantly enhanced the transactivation activity of Tat by up to 2.7 fold (Fig 4A). This synergistic effect was also observed using two patient-derived LTRs (P4T3 and P5T4, n = 3).

We also investigated the effects of HDACi on APOBEC3G induced hypermutated LTRs (defined as a G to A mutation [29]) given that up to ∼1/3 of noninduced proviruses are hypermutated [30]. Despite failing to be activated by Tat alone, these hypermutated sequences were sensitive to reactivation by vorinostat (Fig 4B) and co-stimulation of cells with vorinostat and Tat significantly enhanced the level of luciferase production by up to 7.3-fold (Fig 4B, n = 3).

Finally, given that previous studies have shown that HDACi synergise with prostratin (an NF-κB/PKC activator [31], [32]); we wanted to determine whether this synergism could be demonstrated in this model. Using PMA to activate PKC, we showed that vorinostat and PMA increased the transcriptional activity of NL4-3 LTR-pCEP4 by 1.5 fold (Fig 4C); however, there was no significant synergism between HDACi and PMA using patient-derived HIV LTRs in this model.

## Discussion

HDACi are now being evaluated in HIV-infected patients on suppressive cART as a strategy to eliminate latently infected cells. The response of resting CD4^+^ T-cells from HIV-infected patients on cART to HDACi stimulation is variable both *in vivo* and *ex vivo* but the mechanism for this variable response is currently unclear. Here we demonstrate that the HDACi panobinostat, trichostatin A, vorinostat and entinostat can induce transcription from the majority of HIV LTRs isolated from memory CD4^+^ T-cells from HIV-infected patients on suppressive cART. Furthermore, there were no differences in response to HDACi from HIV LTRs isolated from patients prior to or after treatment with cART. The main factor that determined the magnitude of response to HDACi stimulation was the cellular environment with a maximal fold induction of luciferase observed in the HeLa cell line.

HDACi are subdivided into different classes based on their biochemical structure. Trichostatin A and panobinostat are both hydroxamic acids, whereas vorinostat is a suberoylanilide hydroxamic acid and entinostat is a benzamide derivative [3], [33], [34]. Slight differences in the chemical structure of the various HDACi may affect the way they interact with host histone acetyltransferases and transcription factors. Additionally, panobinostat, trichostatin A and vorinostat are pan-HDACi, whilst entinostat specifically inhibits HDAC1 and 3 [35]. We showed that both vorinostat and panobinostat induced a similar magnitude of luciferase expression when tested at concentrations below the CC_50_. However, panobinostat was substantially more potent (>100 fold) as described in multiple previous studies using CD4^+^ T-cells from HIV-infected patients on cART [13], [36], a primary T-cell model of HIV latency [36], [37] or latently infected cell lines [38], which all showed a significantly lower EC50 of panobinostat compared to vorinostat. The maximum fold response, using concentrations of HDACi that were within the therapeutic range *in vivo* [39] was clearly not dependent on the sequence of the LTR.

The maximum fold increase in luciferase expression induced by HDACi varied significantly between the two cell lines tested, even though there were similar responses to Tat. The differences in response to HDACi between these cell lines were not explained by differential expression of HDACs in these cells or transfection efficiency (Figure S2). The SVG cell line was originally derived from a primary glial cell that is less activated and has lower replication rate and capacity [18] compared to the HeLa cell, a cervical cancer cell, which has a doubling time of approximately 18–24 hours [40]. As most transcription factors are increased in replicating cells or with activation [41], one might expect the transcriptional activity of an SVG to be lower than HeLa, which was indeed what we observed. It is also possible that these two cell lines may differ in other key factors required for transcription of HIV including recruitment of HDAC and histone acetyltransferases to the LTR, and/or the concentration of critical proteins such as positive transcription elongation factor b (P-TEFb) and its association with the inhibitory complex comprising of HEXIM1 and 7SK snRNP [31], [42]–[44]. Phosphorylation of P-TEFb by vorinostat has recently been demonstrated and may also differ between the two cell lines tested in this study [45].

We observed clear synergy between Tat and the HDACi vorinostat – even with LTR sequences that had minimal responsiveness to Tat alone. The activity of Tat is tightly regulated by post-translation modification processes such as acetylation specifically at lysine 28 (K28) and K50/52 [26], [27], [46]. It is possible that vorinostat is capable of modifying Tat's function via acetylation of these key residues. Indeed, synergism between trichostatin A and Tat has previously been reported, and was dependent on lysine residues at K28 and K50 [46]. This synergistic interaction with Tat should be further exploited to increase the activity of HDACi in driving HIV transcription.

Finally, we found that all HDACi tested in this study could activate transcription from the HIV LTRs even when there was evidence of hypermutation. Although these hypermutated LTRs are unlikely to contribute to production of replication competent virus [29], the fact that these hypermutants were sensitive to reactivation by a HDACi could be of biological significance especially if protein produced from these viruses could also stimulate HIV-specific T-cells responses [9].

This is the first study to evaluate the effectiveness of HDACi to activate patient derived LTR sequences *ex vivo* in cells other than T cells. The study highlights the impact of the cellular environment on the ability of HDACi to activate transcription and is important for understanding the use of HDACi in cure strategies. However our approach has several limitations. First, the pCEP system although forming mini chromatin, doesn't integrate into the host genome. It is possible that the nucleosome positioning of these LTRs may therefore be different to what has been observed in HIV-infected cells from patients. However, we believe there are several reasons to expect they would be similar. First, our patient-derived LTR-pCEP4 constructs included all the sequences normally required for integration and nucleosome positioning [47]. Second, it has been shown that nuc-0, 1, 2 are strictly formed at specific positions regardless of the site of virus integration in the host gene [47], [48], suggesting it is the sequence of the LTR that confer nucleosome formation and not the surrounding host or plasmid sequences. However, we recognise that the epigenetic environment of host DNA will also influence the activity of HDACi *in vivo* and the use of an integrating vector with patient derived LTRs would be of interest. These experiments are currently being performed.

In conclusion, we have developed an *ex vivo* model to assess the response of patient derived LTRs to different HDACi. We have shown that changes in the HIV LTR sequence did not translate into differences in sensitivity to activation of transcription by an HDACi. Using concentrations of HDACi close to the CC_50_, similar maximum fold activation was observed for a panel of HDACi. Therefore, we propose that other factors such as the site of integration and the surrounding epigenetic environment are likely to be relevant in determining the variable response of latently infected cells to stimulation with an HDACi.

## Supporting Information

Figure S1 **Dose response of HDACi on the activity of patient-derived HIV LTRs.** HeLa cells was transiently transfected with three patient-derived LTR-pCEP4 and treated with various concentrations of HDACi for 24 hours. The activity of the LTR was measured as the fold change in luciferase compared to the untreated sample. All patient-LTRs produced a similar pattern of response to HDACi compared with the NL4-3 LTR from Figure 3A-II. Error bars represent standard deviation of two independent experiments.
(TIF)

Figure S2 **Expression of HDACs proteins and transfection efficiency of HeLa and SVG cells.** (A) Total cell lysate (20 µg) from HeLa and SVG were probed with antibodies to HDAC1, 2, 3 and 4 and detected by Western blotting. GAPDH was used as a control for equal protein loading. (B) HeLa (left) and SVG cells (right) were transfected with a GFP-expressing plasmid (solid line) or control plasmid (broken line) for 24 hours and expression of GFP was analysed by flow cytometry. Histogram is a representative of two experiments with similar GFP expression.
(TIF)
