# Supplementary figures and images for: Ex Vivo Response to Histone Deacetylase (HDAC) Inhibitors of the HIV Long Terminal Repeat (LTR) Derived from HIV-Infected Patients on Antiretroviral Therapy
Source: PLoS One. 2014 Nov 19;9(11):e113341. doi: 10.1371/journal.pone.0113341 (PMC4237424; doi:10.1371/journal.pone.0113341)

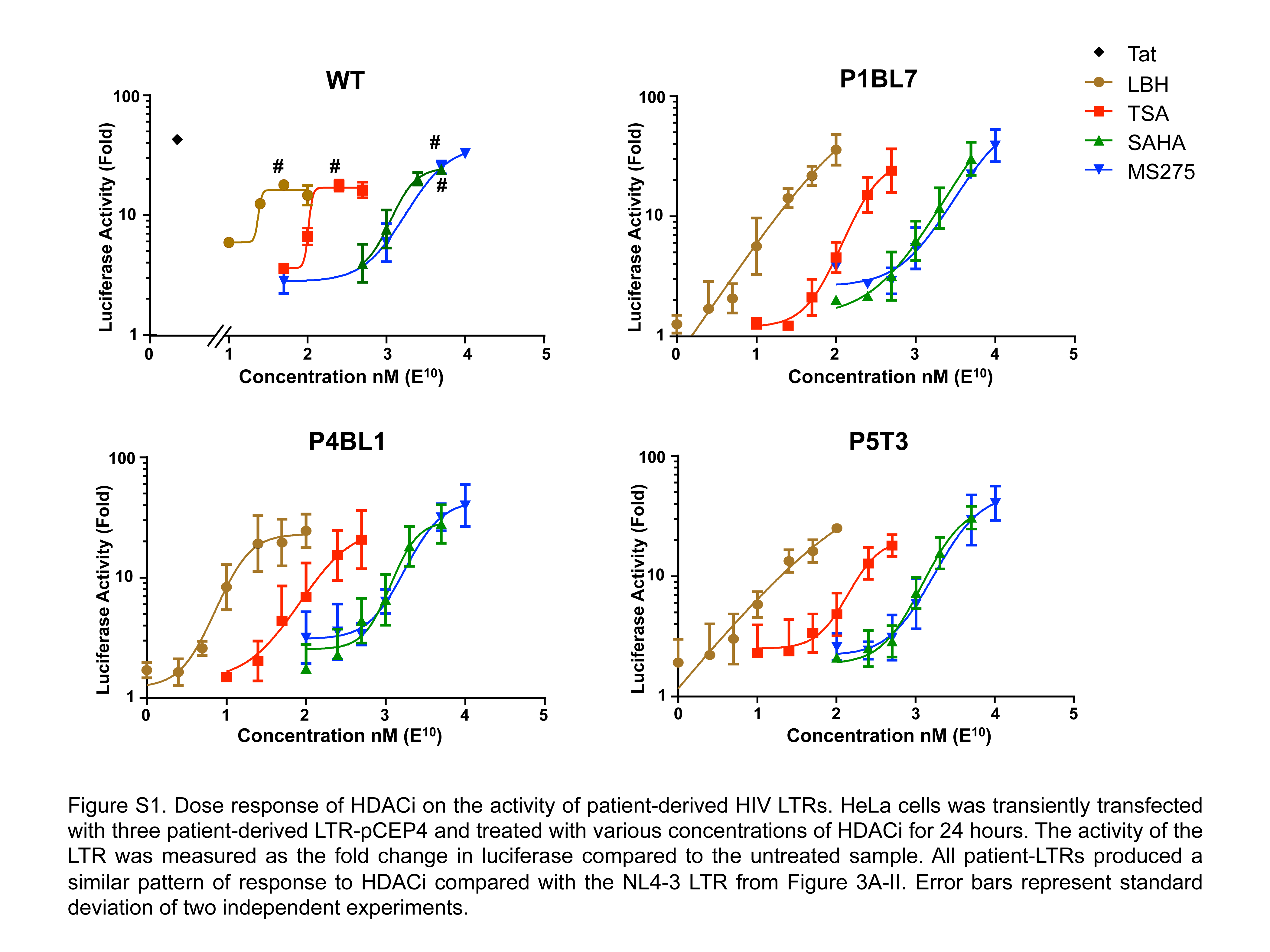

Supplement: Figure S1 — Dose response of HDACi on the activity of patient-derived HIV LTRs. HeLa cells was transiently transfected with three patient-derived LTR-pCEP4 and treated with various concentrations of HDACi for 24 hours. The activity of the LTR was measured as the fold change in luciferase compared to the untreated sample. All patient-LTRs produced a similar pattern of response to HDACi compared with the NL4-3 LTR from Figure 3A-II. Error bars represent standard deviation of two independent experiments. (TIF) [file pone.0113341.s001.tif]

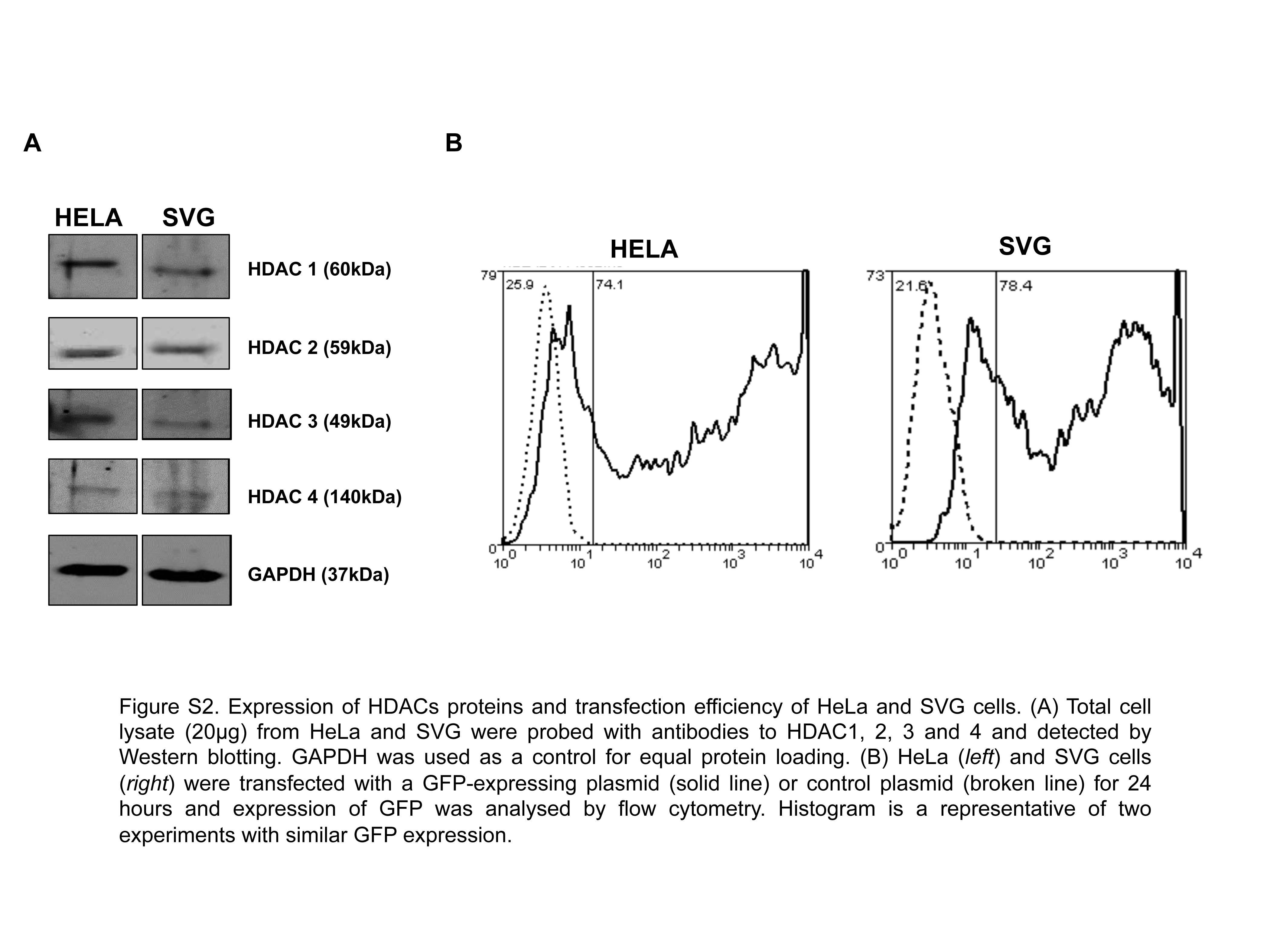

Supplement: Figure S2 — Expression of HDACs proteins and transfection efficiency of HeLa and SVG cells. (A) Total cell lysate (20 µg) from HeLa and SVG were probed with antibodies to HDAC1, 2, 3 and 4 and detected by Western blotting. GAPDH was used as a control for equal protein loading. (B) HeLa (left) and SVG cells (right) were transfected with a GFP-expressing plasmid (solid line) or control plasmid (broken line) for 24 hours and expression of GFP was analysed by flow cytometry. Histogram is a representative of two experiments with similar GFP expression. (TIF) [file pone.0113341.s002.tif]
